# The impact of biceps tenotomy/tenodesis on Popeye sign incidence and functional outcome

**DOI:** 10.1016/j.jseint.2024.10.015

**Published:** 2024-11-23

**Authors:** Farah Selman, Laurent Audigé, Andreas Marc Mueller, ARCR Study Group, Karl Wieser, Florian Grubhofer

**Affiliations:** a Department of Orthopedics, Balgrist University Hospital, University of Zurich, Zurich, Switzerland; b Research and Development, Shoulder and Elbow Surgery, Schulthess Clinic, Zurich, Switzerland; c Surgical Outcome Research Center, Department of Clinical Research, University of Basel c/o University Hospital of Basel, Basel, Switzerland; d Department of Orthopedics and Traumatology, University Hospital, Basel, Switzerland; e ARCR_Pred Study Group, Zurich, Switzerland

**Keywords:** Biceps, Tenotomy, Tenodesis, Popeye, Functional outcome, Rotator cuff repair

## Abstract

**Background:**

Literature reports varied incidences and clinical relevance of Popeye’s sign in patients who underwent biceps tenotomy or tenodesis. There is no consensus according to indication and outcome. We aimed to evaluate the frequency of the Popeye sign in a large cohort of patients concomitantly treated with an arthroscopic rotator cuff repair (ARCR). We assessed the effect on the clinical outcome based on biceps tendon treatment and Popeye sign.

**Methods:**

A cohort of 973 primary ARCR patients from different Swiss and German orthopedic clinics was prospectively documented for up to 24 months postoperatively. All patients who received biceps tendon treatment were included in this study. We assessed Popeye sign occurrence across groups treated either with tenodesis or tenotomy. Additionally, we compared clinical and radiological outcome between Popeye/non-Popeye and tenotomy/tenodesis groups.

**Results:**

Eight hundred patients were evaluated, of which 55% (n = 442) underwent tenodesis and 45% (n = 358) received tenotomy of the long head of the biceps tendon. Mean age of the tenodesis group was significantly lower than that of the tenotomy group (55 ± 9 and 61 ± 8 years, *P* < .001). The other demographics were comparable. Among the patients with tenotomy, 20% (n = 63) developed a Popeye sign, compared to only 6.3% (n = 25) in the tenodesis group (age-adjusted relative risk 3, 95% confidence interval 1.9-4.8; *P* < .001). There were no significant differences in shoulder function. The subjective shoulder value was lower in the Popeye group (82 ± 19% vs. 86 ± 15%, *P* = .010).

**Conclusion:**

Patients with tenotomy of the biceps tendon are 3 times more likely to develop a Popeye sign compared to tenodesis. Popeye sign after ARCR seems to have no relevant effect on the clinical outcome and pain even though the subjective shoulder value was lower in patients with Popeye sign.

The long head of the biceps tendon (LHBT), originating from the supraglenoid tubercle and superior labrum, follows an oblique course toward the intertubercular groove down to the biceps muscle belly.^43^ Its unique anatomical pathway predisposes it to potential abrasion and damage.^43^ In instances of painful LHBT tendinopathy,^35^^,^^45^ surgical interventions such as tenotomy or tenodesis are typically performed, seldom alone, but mostly in combination with a rotator cuff repair.^13^^,^^19^^,^^21^^,^^25^^,^^28^ These procedures, however, can potentially result in a visible deformity known as the Popeye sign, which is characterized by a bulging muscle belly due to tendon detachment and distalization.^41^

The incidence of the Popeye sign in patients following biceps tenotomy or tenodesis is not well documented in large populations.^24^^,^^36^^,^^41^^,^^47^^,^^53^^,^^54^ Some authors assume an incidence of up to 70%.^27^ While typically considered a benign cosmetic issue, some studies have pointed out differences in clinical outcome and patient satisfaction between tenodesis and tenotomy groups, suggesting functional implications that extend beyond aesthetics.^5^^,^^8^^,^^9^^,^^31^^,^^48^^,^^50^ Additionally, there is no consensus in literature according to indication and outcome of biceps tendon tenotomy vs. tenodesis in combination with a rotator cuff repair.^2^^,^^7^^,^^40^

The aim of this study was to evaluate the frequency of the Popeye sign in patients who have undergone biceps tenotomy or tenodesis in a large cohort.^4^ Additionally, we wanted to assess the effect on clinical outcome based on these 2 biceps tendon treatments, as well as the influence of the occurrence of the Popeye sign. The results of this study may help to understand the potential functional implications of these procedures and provide aid in the decision-making process for surgical treatment.

## Methods

### Patient selection and documentation

A cohort of 973 primary arthroscopic rotator cuff repair (ARCR) ^4^ patients were prospectively documented from 19 orthopedic clinics. Data was collected preoperatively and 6, 12, and 24 months postoperatively. All patients who received biceps tendon treatment were included in this analysis. Patients with previous rupture or other prior treatment of the biceps tendon, and patients without documentation of an existing or missing Popeye sign were excluded.

Asymmetry and/or distalization of the muscle belly of the biceps was defined as a positive Popeye sign. The rate of the Popeye sign was assessed and documented by the surgeon and/or supervised orthopedic resident among patients 1 year postoperatively, and its association with functional and quality of life outcomes was investigated. Outcome parameters included range of motion (ROM) (active flexion, abduction and external rotation in 0° abduction; passive internal rotation in 90° abduction), abduction strength, pain level and Constant-Murley Score,^11^ as well as patient-reported subjective evaluation including subjective shoulder value (SSV),^20^ Oxford Shoulder Score (OSS), ^12^ and the EQ-5D-5L utility index.^17^ Subjective outcome parameters were also documented at the 2-year follow-up.

### Surgical techniques and rehabilitation protocol

The operative procedures were performed by trained shoulder surgeons who were all included in the prospective international study. The indication for surgery was reparable rotator cuff tear with or without biceps tendinopathy. After diagnostic arthroscopy and concomitant rotator cuff procedure, proximal tenotomy or tenodesis of the LHBT was performed. The corresponding technique was chosen by the attending surgeon according to his/her preference. All patients underwent a supervised physical therapy program, ordered according to the concomitant procedures on the rotator cuff.

### Data management and statistical analysis

Baseline and clinical collected data were managed using the REDCap electronic data capture system (Vanderbilt University, Nashville, TN, USA) ^22^ and exported for statistical analysis using Intercooled Stata, version 17 (StataCorp LP, College Station, TX, USA). Baseline patient parameters were tabulated separately per LHBT treatment group using standard descriptive statistics and compared using standardized differences (StdDiff) (where values closest to 0.10 indicate stronger group similarity) and clinical judgment.

The presence of a Popeye sign at 12 months was tabulated by study group with absolute and relative frequencies (risk) and compared between biceps treatment groups using binomial regression adjusted for the patient age at surgery. The strength of association was reported in term of relative risk (RR) along with its 95% confidence interval (CI). We compared baseline patient parameters between patients with a Popeye sign and patients without to identify factors likely to confound any association between Popeye sign and outcome. Continuous outcome parameters were compared between these 2 groups using regression analysis adjusted for the patient age, sex, biceps treatment, and baseline parameter values.

We compared continuous outcome parameters between biceps treatment groups using generalized linear mixed models to account for repeated measurements at each follow-up time point up to 24 months follow-up. All models were adjusted for the patient age at surgery. Regression coefficients (beta) were estimated along with their 95% CI. All analyses were explorative with a significance level set at 0.05.

Sample size was not a priori estimated for this analysis, as all eligible patients were selected from the ARCR_Pred cohort. Nevertheless, given our sample size in each group, from a baseline risk of 20% in the tenotomy group, this analysis had 95% power to identify a 50% risk reduction of Popeye sign at the 12-month follow-up time point when using biceps tenodesis, at a significance level of 5%.

## Results

From the whole ARCR_Pred cohort study,^4^ 877 patients had nonruptured or nontreated biceps tendon, as observed intraoperatively, 800 (91%) of which were treated either by tenotomy (n = 358) or tenodesis (n = 442). The follow-up rate at 12 months was 90%, consisting of 719 patients.

### Tenotomy vs. tenodesis

The baseline demographics for tenotomy and tenodesis group were comparable according to sex, dominant side, body mass index (BMI), rotator cuff tear pattern (combinations) and severity (Table I). Patients treated with tenotomy showed a higher mean age compared to the tenodesis group (61 ± 8 years vs. 55 ± 9 years, StdDif 0.628). There were various tenodesis techniques documented in the ARCR cohort, including 49% (n = 218) were performed suprapectoral with an anchor, and 46% (n = 204) were subpectoral, of which 52% (n = 105) were fixed with an anchor and 47% (n = 96) with a cortical button.

**Table I:** Baseline parameters tenotomy vs. tenodesis.

Total N = 800	Tenodesis N = 442 (55%)	Tenotomy N = 358 (45%)	Standardized mean difference
Mean age [yrs]	55 ± 9	61 ± 8	0.628∗
Gender [female]	145 (33%)	154 (43%)	0.212
Side [dominant]	320 (72%)	257 (72%)	0.014
BMI [kg/m^2^]	26 ± 4	28 ± 5	0.315
RC tendons ruptured (SSP, ISP, SSC)			0.274
RC tear severity (partial, full, massive)			0.282
Tear profile (combinations)			0.367

*BMI*, body mass index; *RC*, rotator cuff; *SSP*, supraspinatus tendon; *ISP*, infraspinatus tendon; *SSC*, subscapularis tendon.

All values are given in means (Standard deviation).

∗ Marks statistical significance.

### Popeye sign

Twenty percent (n = 63) of patients with tenotomy developed a Popeye sign, whereas only 6.2% (n = 25) of the tenodesis group showed a visible dislocated muscle belly. Patients with tenotomy were 3.1 times as likely to report a Popeye sign than if a tenodesis was performed (age-adjusted RR, *P* < .001, 95% CI 1.9-4.8).

Male have more often Popeye sign than females (n = 74, 16% vs. n = 14, 5%). Male show a significant RR of 3.5% (95% CI 2.1-6.1) in the binominal regression analysis. BMI does not show a significant association, but there is a tendency for obese patients not to show a Popeye sign compared to normal patients.

There were no statistically significant differences between the Popeye and non-Popeye group at the 1-year follow-up for most shoulder function and quality of life parameters (Table II). Patients with a Popeye sign showed a statistically significantly lower active shoulder abduction by 7° (95% CI 1-12; *P* = .016), lower SSV by 4% (95% CI 1-8; *P* = .008), and lower OSS by 2 points (95% CI 0-3; *P* = .045).

**Table II:** Outcome Popeye vs. Non-Popeye at 1-year follow-up.

Outcome measure	No popeye mean (SD)	Popeye mean (SD)	*P* value
Flexion active °	159 (21)	154 (25)	.073
Mean strength kg	6.0 (3.0)	6.1 (3.6)	.900
Relative Constant score 0-100	77 (12)	76 (15)	.165
EQ-5D-5L utility index	0.92 (0.13)	0.91 (0.18)	.609
Subjective shoulder value %	86 (15)	82 (19)	.010∗
Pain level 0-10	1.2 (1.9)	1.2 (1.9)	.875

*SD*, standard deviation; *EQ-5D-5L*, EuroQol 5-dimension 5-level.

All values are given in means (Standard deviation).

∗ Marks significance of the *P* value.

### Biceps treatment and patient outcomes

Across all follow-up time points, there were no overall significant differences in active flexion, active abduction, passive internal rotation in 90° abduction, pain level, OSS, and quality of life EQ-5D-5L utility index (Tables III and IV). Active external rotation showed statistically significant but not clinically relevant differences of 3-4 degrees. There were significant differences in abduction strength of about 1 kg at 6- and 12-month follow-up, favorizing the tenodesis group (*P* < .001). Statistically significant, but not clinically relevant differences of 3 points in the constant score (CS) could be observed at the 12-month follow-up (*P* < .001); a group difference of 1% in the SSV was also observed at 12 months (*P* = .397).

**Table III:** Clinical outcome tenotomy vs. tenodesis at 2-year follow-up.

Outcome measure	Tenodesis	Mean (SD)	Tenotomy	Mean (SD)	*P* value	Mixed model
	n		n			*P* value
Flexion active [°]						
Baseline	442	130 (45)	358	123 (44)		.472
6 mo	420	148 (27)	340	144 (29)	.091	
12 mo	402	159 (20)	315	157 (24)	.144	
Change baseline to 6 mo	420	17 (42)	340	20 (44)	.288	
Change baseline to 12 mo	402	29 (43)	315	32 (43)	.264	
Abduction active [°]						
Baseline	442	116 (48)	358	114 (45)		.200
6 mo	420	142 (32)	340	138 (34)	.147	
12 mo	402	156 (23)	315	154 (27)	.159	
Change baseline to 6 mo	420	26 (48)	340	25 (49)	.776	
Change baseline to 12 mo	402	41 (48)	315	39 (47)	.629	
External rotation in 0° abduction, active [°]						
Baseline	442	48 (19)	358	47 (19)		.002∗
6 mo	421	47 (17)	333	50 (18)	.014∗	
12 mo	401	54 (17)	315	57 (16)	.012∗	
Change baseline to 6 mo	421	−2 (20)	333	2 (21)	.006∗	
Change baseline to 12 mo	401	5 (20)	315	9 (20)	.012∗	
Internal rotation in 90° abduction, passive [°]						
Baseline	441	36 (25)	358	39 (25)		.269
6 mo	420	38 (25)	330	40 (24)	.227	
12 mo	401	44 (25)	314	47 (25)	.063	
Change baseline to 6 mo	420	2 (27)	330	2 (32)	.879	
Change baseline to 12 mo	401	8 (27)	314	8 (32)	.891	
Abduction strength [kg]						
Baseline	441	3.3 (3.2)	358	2.7 (2.7)		<.001∗
6 mo	409	5.0 (3.0)	326	4.0 (2.7)	<.001∗	
12 mo	395	6.7 (3.1)	309	5.3 (2.8)	<.001∗	
Change baseline to 6 mo	408	1.7 (3.5)	326	1.3 (3.0)	.064	
Change baseline to 12 mo	394	3.4 (3.5)	309	2.5 (3.3)	<.001∗	
Pain level [0-10]						
Baseline	442	5.5 (2.3)	358	5.5 (2.3)		.520
6 mo	421	1.9 (2.1)	339	1.7 (2.1)	.393	
12 mo	402	1.2 (2.0)	316	1.2 (1.8)	.783	
Change baseline to 6 mo	421	−3.6 (2.8)	339	−3.8 (2.8)	.284	
Change baseline to 12 mo	402	−4.3 (2.8)	316	−4.3 (2.5)	.722	
Constant score, relative						
Baseline	441	51 (19)	358	48 (18)		.009∗
6 mo	409	69 (16)	326	67 (15)	.051	
12 mo	394	79 (11)	309	76 (12)	<.001∗	
Change baseline to 6 mo	408	18 (20)	326	19 (19)	.534	
Change baseline to 12 mo	393	28 (19)	309	27 (18)	.498	

*SD*, standard deviation.

All values are given in means (Standard deviation SD).

∗ Marks significance of the *P* value.

**Table IV:** PROM tenotomy vs. tenodesis at 2-year follow-up.

	Tenodesis	Mean (SD)	Tenotomy	Mean (SD)	*P* value
	n		n		
OSS [0-48]					
Baseline	442	27 (10)	358	27 (8)	
6 mo	421	39 (8)	331	40 (8)	.071
12 mo	410	43 (6)	313	43 (7)	.326
24 mo	367	44 (6)	288	44 (7)	.478
Change baseline to 6 mo	421	12 (9)	331	13 (9)	.140
Change baseline to 12 mo	410	16 (9)	313	16 (9)	.610
Change baseline to 24 mo	367	17 (9)	288	17 (9)	.543
SSV [%]					
Baseline	442	46 (20)	358	46 (19)	
6 mo	421	76 (18)	330	76 (19)	.881
12 mo	409	86 (15)	313	85 (16)	.397
24 mo	368	89 (16)	286	88 (16)	.531
Change baseline to 6 mo	421	30 (25)	330	29 (23)	.735
Change baseline to 12 mo	409	40 (23)	313	39 (24)	.470
Change baseline to 24 mo	368	43 (24)	286	42 (23)	.375
EQ-5D-5L					
Baseline	442	0.70 (0.24)	358	0.70 (0.23)	
6 mo	421	0.88 (0.14)	331	0.89 (0.14)	.188
12 mo	410	0.92 (0.11)	313	0.91 (0.15)	.248
24 mo	367	0.93 (0.12)	285	0.93 (0.13)	.849
Change baseline to 6 mo	421	0.17 (0.22)	331	0.19 (0.22)	.451
Change baseline to 12 mo	410	0.21 (0.22)	313	0.21 (0.22)	.771
Change baseline to 24 mo	367	0.22 (0.22)	285	0.21 (0.21)	.567
Pain VAS [0-100]					
Baseline	442	67 (20)	358	69 (19)	
6 mo	421	79 (17)	331	81 (16)	.153
12 mo	410	84 (16)	313	84 (15)	.970
24 mo	368	85 (16)	288	84 (16)	.297
Change baseline to 6 mo	421	11 (19)	331	11 (20)	.873
Change baseline to 12 mo	410	16 (20)	313	14 (21)	.330
Change baseline to 24 mo	368	17 (21)	288	14 (22)	.189

*PROM*, patient reported outcome measure; *SD*, standard deviation; *OSS*, Oxford Shoulder Scale; *SSV*, subjective shoulder value; *EQ-5D-5L*, EuroQol 5-dimension 5-level; *VAS*, visual analog scale.

All values are given in means (Standard deviation).

## Discussion

The current study aimed to assess the incidence of the Popeye sign in patients who underwent biceps tenotomy or tenodesis, as well as to evaluate its impact on clinical outcomes. Additionally, a comparison of functional and subjective outcomes between a tenotomy and tenodesis group was performed.

A positive Popeye sign indicates a rupture of the proximal LHBT with distalization of the biceps brachii muscle belly.^16^ We have therefore assumed that Popeye sign incidence is higher in patients treated with tenotomy compared to tenodesis, in which the stump of the tendon is refixed further distally. As expected and shown several times in literature,^10^^,^^13^^,^^24^^,^^34^ there were more Popeye signs in the tenotomy group compared to the tenodesis group (20% vs. 6%). The RR of developing a Popeye sign in the tenotomy group was 3 times as high as in the tenodesis group (age adjusted RR 3, 95% CI 1.9-4.8). This result is comparable to Woodmass’s randomized clinical trial,^51^ which reported 4.3 higher odds for biceps deformity in the tenotomy group and systematic review from Liu et al,^34^ which showed RRs between 2.4 and 3.3.

Nevertheless, not all tenotomy patients have shown a Popeye sign. This could be due to incorrect diagnosis by the surgeon, but more likely by muscle status and soft tissue coverage, which could play a role in the development and visibility of a Popeye deformity. Almeida et al ^3^ suggest to perform a tenodesis in patients with a BMI < 30, as these patients seem to be more complaining about esthetic deformity compared to the heavier population. We could not show a significant association, but a tendency for obese patients not to show or report a Popeye sign. Kelly et al ^27^ showed a higher rate of Popeye sign in males compared to females (82.7% vs. 36.5%). They concluded that the difference is caused by sex-related variability in muscle status. In our population, males showed a significant higher rate of Popeye compared to females with a RR of 3.5. A group from Lebanon^16^ used a radiopaque device before tenotomy to measure biceps tendon retraction over time. They showed a dynamic dislocation from day 1 to day 90 after tenotomy. This finding could additionally lead to missed Popeye deformities during early examination. All these factors could possibly additionally explain the high variability on Popeye sign incidences in the literature which vary from 14.1% up to 70%.^10^^,^^13^^,^^27^^,^^38^ Our incidence of 20% in the tenotomy group is within the lower range.

The overall incidence of a Popeye sign in our tenodesis group amounts to 6.3%. Analog to the rates of Popeye sign after tenotomy, the incidence varies in a wide range among different authors.^10^^,^^32^^,^^39^ The development of a Popeye sign in the tenodesis patients could be caused by a secondary dislocation of the muscle after fixation failure or due to a distalized reattachment of the corresponding tendon. McCrum et al ^37^ report a failure rate of 0.8% (rerupture, failure of fixation of the tendon to bone) after tenodesis.

According to clinical outcome in patients with and without Popeye sign, there were no relevant differences at the 1-year follow-up for active flexion, mean abduction strength, relative CS, EQ-5D-5L utility index and pain level. The SSV showed significant difference in statistical testing, but a 4% difference is expected not to be relevant for the quality of life.^26^ Overall, patients seem to be satisfied with the procedure, independent of the muscle deformity, shown by a postoperative SSV ≥ 82% in both groups. These results are comparable to those of Pouliquen et al^41^ who reported no significant clinical differences in patients with and without Popeye sign. Boileau et al^8^ identified 24 patients with a Popeye sign after LHBT surgery. Sixty-seven percent of those patients had noticed the deformity, but none of them were bothered by its presence. Koh et al^28^ reported a similar observation with 17% of patients with Popeye deformity. None of these were unsatisfied or bothered by it either. Whether a postoperative asymmetry on the upper arm is recognized or not, may be dependent on the age and sex of the patient.^33^^,^^38^ Duff and Campbell^14^ investigated acceptance of LHBT tenotomy in 117 younger patients with a Popeye sign incidence of 57%, of which 11% were bothered by it. There are indications in literature, that older patients tend to pay less attention to an upper arm deformity than the younger population and thereby be less bothered by it.^8^^,^^14^

We could not show clinically relevant differences in the ROM or CS between the tenodesis and tenotomy group which is consistent with existing systematic reviews and meta-analyses.^2^^,^^6^^,^^23^^,^^29^^,^^42^^,^^53^ Only a few studies favorize tenotomy over tenodesis.^5^^,^^52^ Yogun et al^52^ showed no differences for clinical score, but a higher bicipital groove tenderness and muscle cramping in the tenodesis group. Belay et al^5^ observed also no functional differences but an earlier improvement in postoperative pain in the tenotomy group. We could observe higher abduction forces at baseline, 6- and 12-month postoperative in the tenodesis group. As the LHBT has no relevant influence on abduction force, these differences are more likely due to younger mean age in the tenodesis group with expected higher strength.

### Limitations

Our cohort showed a significant standardized mean difference in age between the tenotomy and tenodesis group (61 ± 8 years vs. 55 ± 9 years). This finding is recognized as a bias of our comparability, however we adjusted for age in all regression models. This observation is consistent with the literature, where older patients have received a tenotomy more frequently than younger patients.^1^^,^^5^^,^^9^^,^^25^

Our cohort consisted of patients with ARCR^4^ and a concomitant treatment of the LHBT, not an isolated biceps tendon treatment. While the intervention on the rotator cuff could influence our results, LHBT injuries often appear, and therefore are often treated together with rotator cuff tears.^15^^,^^18^^,^^43^^,^^44^^,^^46^^,^^49^ The association of an instability of the LHBT with a subscapularis tendon tear has been investigated frequently.^30^^,^^46^

Due to the ARCR_Pred cohort study design with multiple clinical objectives toward the cuff tear repairs themselves, we could not examine our population biceps tendon–specific. Fundamental clinical parameters which correlate directly with the force of the biceps, like elbow flexion and forearm supination could not be measured. Exploring the potential correlation between the Popeye sign and the loss of strength would have been interesting to investigate, especially considering Wittenstein’s findings with significantly greater decrease in supination strength following tenotomy as compared to tenodesis.^50^

## Conclusion

Patients with tenotomy of the biceps tendon are 3 times more likely to develop a Popeye sign compared to tenodesis. Popeye sign seems to have no relevant effect on the Constant score or SSV. ARCR patients with tenotomy or tenodesis can expect similar postoperative shoulder function and pain levels.

## Acknowledgment

The authors thank ARCR_Pred study team members for their valuable engagement in all aspect of study implementation. Members of the ARCR_Pred Study Group are listed below per site and partner institution: ARTHRO Medics, Basel, CH (ART): Claudio Rosso (Principal Investigator [PI]); Charitè Medicine University, Berlin, DE (BER): Philipp Moroder (PI), Doruk Akgün, Isabella Weiss, Eduardo Samaniego; Cantonal Hospital Baselland, Bruderholz, CH (BRU): Thomas Suter (PI), Sebastian A. Müller, Markus Saner, Claudia Haag-Schumacher; Public Hospital Solothurn, Solothurn, CH (BSS): Mai Lan Dao Trong (PI), Carlos Buitrago-Tellez, Julian Hasler, Ulf Riede; Hôpital du Valais –Centre Hospitalier du Valais Romand, Martigny, CH (CHV): Beat Moor (PI), Matthias Biner, Nicolas Gallusser; Endoclinic, Zurich, CH (END): Christoph Spormann (PI), Britta Hansen; Klinik Gut, St Moritz, CH (GUT): Holger Durchholz (PI); Hirslanden Clinique la Colline, Geneva, CH (HIR): Gregory Cunningham (PI); La Tour Hospital, Meyrin, CH (HUG): Alexandre Lädermann (PI); Inselspital, Bern, CH (INB): Michael Schär (PI), Rainer Egli, Stephanie Erdbrink, Kate Gerber, Paolo Lombardo, Johannes Weihs; In-Motion, Wallisellen, CH (INM): Matthias Flury (PI), Ralph Berther, Christine Ehrmann, Larissa Hübscher; Institute of Social and Preventive Medicine (ISPM), University Bern, Bern, CH: David Schwappach; Cantonal Hospital Baden, Baden, CH (KSB): Karim Eid (PI), Susanne Bensler, Yannick Fritz; Cantonal Hospital Winterthur, Winterthur, CH (KSW): Emanuel Benninger (PI), Philemon Grimm, Markus Pisan; Schulthess Klinik, Zurich, CH (KWS): Markus Scheibel (PI), Laurent Audigé, Daniela Brune, Marije de Jong, Stefan Diermayr, Marco Etter, Florian Freislederer, Michael Glanzmann, Cécile Grobet, Christian Jung, Fabrizio Moro, Ralph Ringer, Jan Schätz, Hans-Kaspar Schwyzer, Martina Wehrli, Barbara Wirth; Ospedale Regionale di Lugano, Lugano, CH (LUG): Christian Candrian (PI), Filippo Del Grande, Pietro Feltri, Giuseppe Filardo, Francesco Marbach, Florian Schönweger; Cantonal Hospital St. Gallen, St. Gallen, CH (SGA): Bernhard Jost (PI), Michael Badulescu, Stephanie Lüscher, Fabian Napieralski, Lena Öhrström, Martin Olach, Jan Rechsteiner, Jörg Scheler, Christian Spross, Vilijam Zdravkovic; Orthopädie Sonnenhof, Bern, CH (SON): Matthias A. Zumstein (PI), Annabel Hayoz, Julia Müller-Lebschi; University Clinic Balgrist, Zurich, CH (UKB): Karl Wieser (PI), Paul Borbas, Samy Bouaicha, Roland Camenzind, Sabrina Catanzaro, Christian Gerber, Florian Grubhofer, Anita Hasler, Bettina Hochreiter, Roy Marcus, Farah Selman, Reto Sutter, Sabine Wyss; University Library Basel, University Basel, Basel, CH: Christian Appenzeller-Herzog; University Hospital Basel, Basel, CH (USB): Andreas Marc Müller (PI), Soheila Aghlmandi, Cornelia Baum, Franziska Eckers, Kushtrim Grezda, Simone Hatz, Sabina Hunziker, Thomas Stojanov, Mohy Taha, Giorgio Tamborrini-Schütz.

## Disclaimers:

Funding: This project is funded by the Swiss National Science Foundation (SNF Project ID 320030_184959, http://p3.snf.ch/project-184959). A complementary grant was provided by Swiss Orthopedics to support project site documentation. The following sites: Charitè Medicine University, Berlin, Germany (BER); Public Hospital Solothurn, Solothurn, Switzerland (BSS); Endoclinic, Zurich, Switzerland (END); Inselspital, Bern, Switzerland (INB); and University Clinic Balgrist, Zurich, Switzerland (UKB) are funding their own participation in the project.

Conflicts of interest: The authors, their immediate families, and any research foundations with which they are affiliated have not received any financial payments or other benefits from any commercial entity related to the subject of this article.
